# Functional Characterization of *TaNCED1* in Regulating Drought Stress Tolerance in Transgenic Wheat

**DOI:** 10.3390/plants15162415

**Published:** 2026-08-07

**Authors:** Shujuan Zhang, Rongzhi Zhang, Dandan Li, Jie Gao, Jihu Li, Guoqi Song, Wei Li, Yulian Li, Genying Li

**Affiliations:** 1 Crop Research Institute, Shandong Academy of Agricultural Sciences, Jinan 250100, China; 2 State Key Laboratory of Wheat Improvement, Jinan 250100, China; 3 National Engineering Research Center for Wheat and Corn, Jinan 250100, China; 4 Key Laboratory of Wheat Biology and Genetic Improvement in the Northern Yellow and Huai River Valley, Ministry of Agriculture and Rural Affairs, Jinan 250100, China; 5 College of Agronomy, Shandong Agricultural University, Tai’an 271018, China

**Keywords:** NCED, CRISPR, common wheat, drought stress, membrane stability, RWC

## Abstract

The ABA signaling pathway is essential for plant growth, development, and responses to diverse abiotic stresses. 9-cis-epoxycarotenoid dioxygenases (NCEDs) are key functional enzymes involved in this pathway. In the present study, we investigated the biological roles and underlying molecular mechanisms of the *TaNCED1* gene in wheat under drought stress treatment, using two materials: *TaNCED1*-overexpressing transgenic wheat plants and the *nced1* knockout mutants. Phenotypic and physiological results demonstrated that *TaNCED1* overexpression significantly enhanced wheat drought tolerance, whereas the knockout mutants clearly reduced the drought resistance. Mechanistically, *TaNCED1* positively modulates wheat drought tolerance by maintaining higher leaf relative water content (RWC), accumulating more soluble sugars and proline, and reducing malondialdehyde (MDA) accumulation and electrolytic leakage. Moreover, the catalase (CAT) activity accumulated to higher levels exclusively under drought stress, and this antioxidant enzyme serves as a core mediator of drought tolerance conferred by *TaNCED1* overexpression. Transcriptomic profiling of drought-challenged transgenic and WT plants further confirmed that *TaNCED1* initiates a suite of stress-responsive regulatory cascades and robustly upregulates key drought resistance genes in wheat.

## 1. Introduction

ABA plays important roles in regulating plant growth and development. Endogenous ABA content in plant tissues is precisely modulated by its biosynthetic pathway. Core genes associated with ABA biosynthesis have been successfully cloned and identified, including zeaxanthin epoxidase (ZEP), 9-cis-epoxycarotenoid dioxygenase (NCED), and abscisic aldehyde oxidase (AAO). In higher plants, NCEDs are widely recognized as rate-limiting enzymes controlling ABA biosynthesis and play indispensable roles in plant stress tolerance [1,2]. The first *NCED* gene, *VP14*, was isolated and characterized in maize [3]. Subsequently, homologous *NCED* genes have been isolated from a wide range of plant species, including tomato [4], avocado [5], *Arabidopsis* [6,7], *Malus* [8], and *Brassica napus* [9].

Extensive research has clarified the vital roles of ABA in modulating plant growth and adaptation to abiotic stresses. Accumulating evidence has demonstrated that elevated *NCED* transcript levels could promote ABA biosynthesis and increase ABA accumulation in plants. Ectopic expression of *NCED1* increases the endogenous ABA levels, reduces leaf transpiration, and thereby enhances plant drought tolerance [1]. Consistently, overexpression of the *NCED3* and *NCED6* in *Arabidopsis* [10] and heterologous expression of the *NCED1* of bean (*PvNCED1*), tomato (*LeNCED1*), and *Citrus reshni* (*CrNCED1*) in tobacco [11,12,13] have all been proven to boost leaf ABA accumulation and strengthen drought resistance. Notably, different *NCED* family genes exhibit distinct response patterns to specific stress stimuli, suggesting functional divergence among *NCED* members.

In our previous work, a wheat *NCED* family gene was isolated via homologous cloning and preliminarily named as *TaNCED1*. Heterologous research in tobacco has implied its potential participation in abiotic stress response pathways [14]. Nevertheless, the endogenous biological functions and regulatory mechanisms of this gene in wheat under stress have not been systematically explored. And the physiological roles of wheat *NCED* family genes in stress adaptation remain largely underexplored. However, the biological functions and underlying regulatory mechanisms of *TaNCED1* in wheat abiotic stress responses remain uncharacterized, and its endogenous physiological roles in wheat have not yet been elucidated. Overall, the stress-responsive physiological mechanisms of *NCED* genes in wheat are still poorly understood.

Common wheat is one of the most crucial staple food crops in the world. Drought stress is a major environmental constraint that severely limits wheat yield and quality. Therefore, exploring the functions of drought-responsive genes and dissecting their molecular regulatory mechanisms is of great significance for mining elite drought-resistant genetic resources and accelerating molecular breeding of drought-tolerant wheat varieties. Given that NCED acts as the rate-limiting enzyme in the ABA biosynthesis pathway, it is a core candidate gene for crop stress resistance improvement. As the endogenous role of *TaNCED1* in wheat has not been fully elucidated, *TaNCED1*-overexpressing lines and *nced1* knockout mutants were employed to systematically explore its physiological roles under drought stress. Phenotypic and physiological analyses revealed that *TaNCED1* overexpression significantly enhanced wheat drought tolerance, whereas the knockout mutants markedly decreased drought resistance in wheat.

## 2. Results

### 2.1. Generation of TaNCED1 Transgenic Wheat Lines

To elucidate the function of *TaNCED1* in regulating drought tolerance in wheat, we generated two types of wheat genetic materials, including *TaNCED1*-overexpression (OE) and CRISPR/Cas9-mediated knockout mutants, via the *Agrobacterium*-mediated wheat genetic transformation system [15].

**Figure 1:**
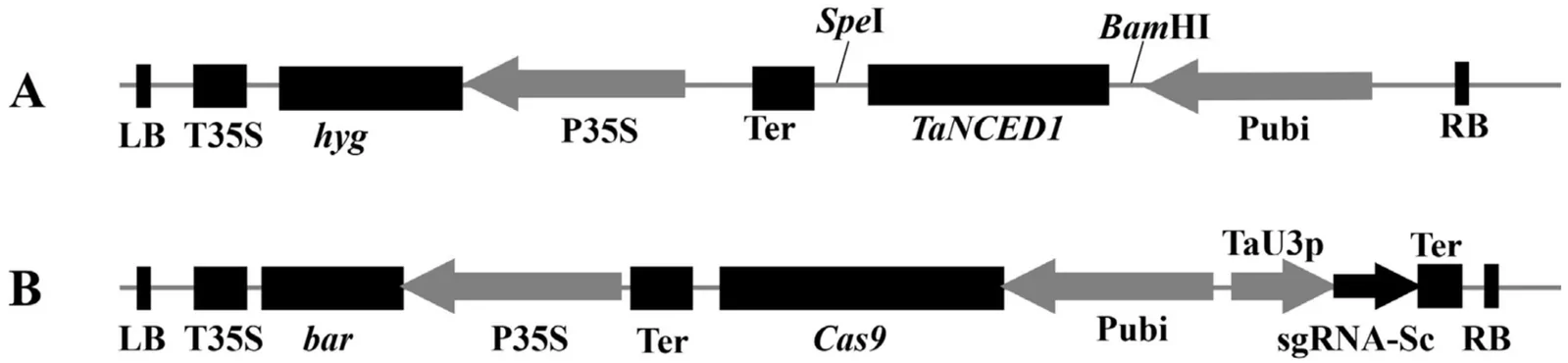
Schematic diagram of the T-DNA region of the binary vector. (**A**) T-DNA region of the *TaNCED1* overexpression vector. LB, Left border. RB, Right border. Pubi, maize ubiquitin promoter. *TaNCED1*, 9-cis-epoxycarotenoid dioxygenases gene from *Triticum aestivum* L.; *hyg*, hygromycin B resistance gene. (**B**) T-DNA structure of the *TaNCED1* knockout vector, Cas9 was expressed with a ubiquitin promoter, and the sgRNA was derived using U3 promoters.

For the construction of overexpression vector, the ubiquitin promoter was employed to initiate the transcription of *TaNCED1*. A hygromycin resistance gene (*hyg*) was driven by the CaMV35S promoter, which enabled the screening of positive transgenic lines through hygromycin B screening (Figure 1).

The CRISPR/Cas9 gene-editing system was utilized to generate *nced1* knockout mutants. Given that *TaNCED1* has three homoeologous copies on wheat subgenome 5A (TraesFLD5A01G034600.1), 5B (TraesFLD01G032700.1) and 5D (TraesFLD5D01G045900.1), a specific single-guided RNA (sgRNA) was designed to target the conserved sites of all three homoeologs. In the editing vector, the wheat U3 promoter and maize ubiquitin promoter were separately employed to drive the expression of sgRNA and Cas9 protein. Additionally, the vector carried a herbicide resistance *Bar* gene, allowing positive mutant screening through Basta herbicide selection.

### 2.2. Molecular Characterization of the Transgenic Plants

**Figure 2:**
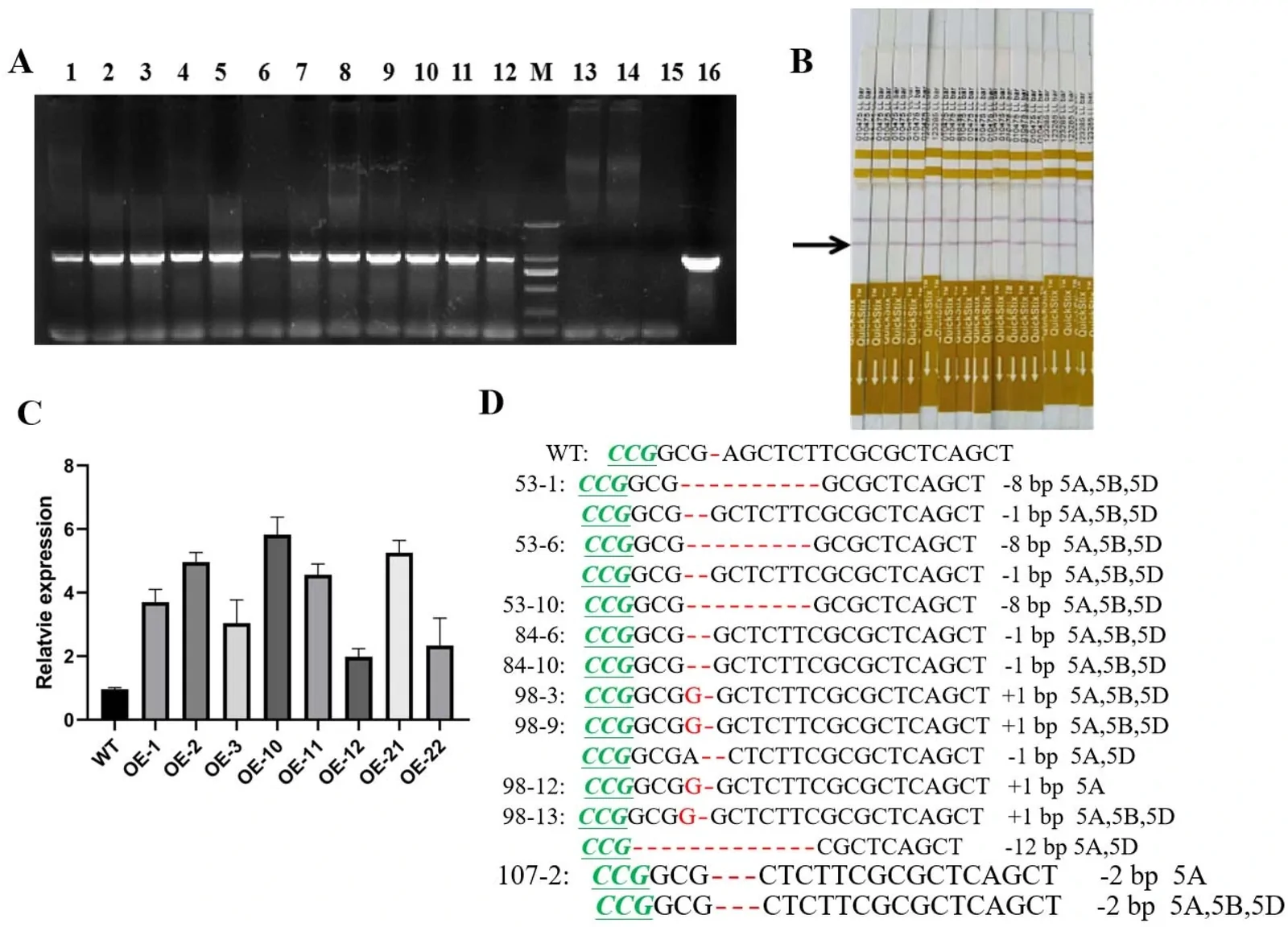
Molecular characterization of transgenic wheat plants. (**A**) PCR identification of independent transgenic lines using the *hyg* marker gene.Lanes 1–14 correspond to independent transgenic lines; Lane 15 is the negative control, and Lane 16 is the positive control. (**B**) Bar protein detection of transgenic wheat plants. The arrow‑pointed band confirms the presence of positive‑plant bands. (**C**) Relative expression analysis of the *TaNCED1* gene in transgenic wheat lines. (**D**) CRISPR/Cas9-induced targeted mutagenesis of the *TaNCED1* gene. The mutation sites are marked in red. The PAM (NGG) sites are underlined and highlighted in green italics.

Genomic identification was initially performed to screen candidate transgenic lines. PCR amplification of the *hyg* gene confirmed the integration of exogenous fragments into the genomes of OE lines. Clear and specific bands were present in the transgenic lines, while no amplification products were observed in WT plants (Figure 2A). Primary screening of CRISPR/Cas9-mediated *nced* mutants was carried out using a QuickStix kit for rapid *Bar* protein detection (Figure 2B).

Total RNA was isolated from the leaves of transgenic wheat, and quantitative real-time RT-PCR was performed to examine *TaNCED1* expression. Significant variations in *TaNCED1* expression were observed among different transgenic lines. As presented in Figure 2C, OE lines exhibited substantially upregulated *TaNCED1* expression relative to WT plants. The exact mutation types of *nced* knockout lines were further identified using the Hi-TOM mutation detection kit (Figure 2D). Genomic fragments flanking the sgRNA target sites were amplified by PCR with gene-specific primers. Sequencing analysis revealed multiple mutation patterns, including 1 bp deletion (1d), 1 bp insertion (1i), 2 bp deletion (2d), and 8 bp deletion (8d).

Molecular characterization was performed on transgenic plants in each generation. For overexpression lines, transgene integration and transcript abundance were examined. For gene-edited lines, mutation type and editing efficiency were detected. Homozygous transgenic plants were identified, selected, and subjected to preliminary screening for drought tolerance at the seedling stage. Homozygous lines exhibiting evident drought-resistant phenotypes in preliminary assays were selected for subsequent physiological and phenotypic experiments. Homozygous lines of OE and *nced1* mutants were finally screened and selected for subsequent functional experiments.

### 2.3. Overexpression of TaNCED1 Significantly Improves Drought Tolerance in Wheat

**Figure 3:**
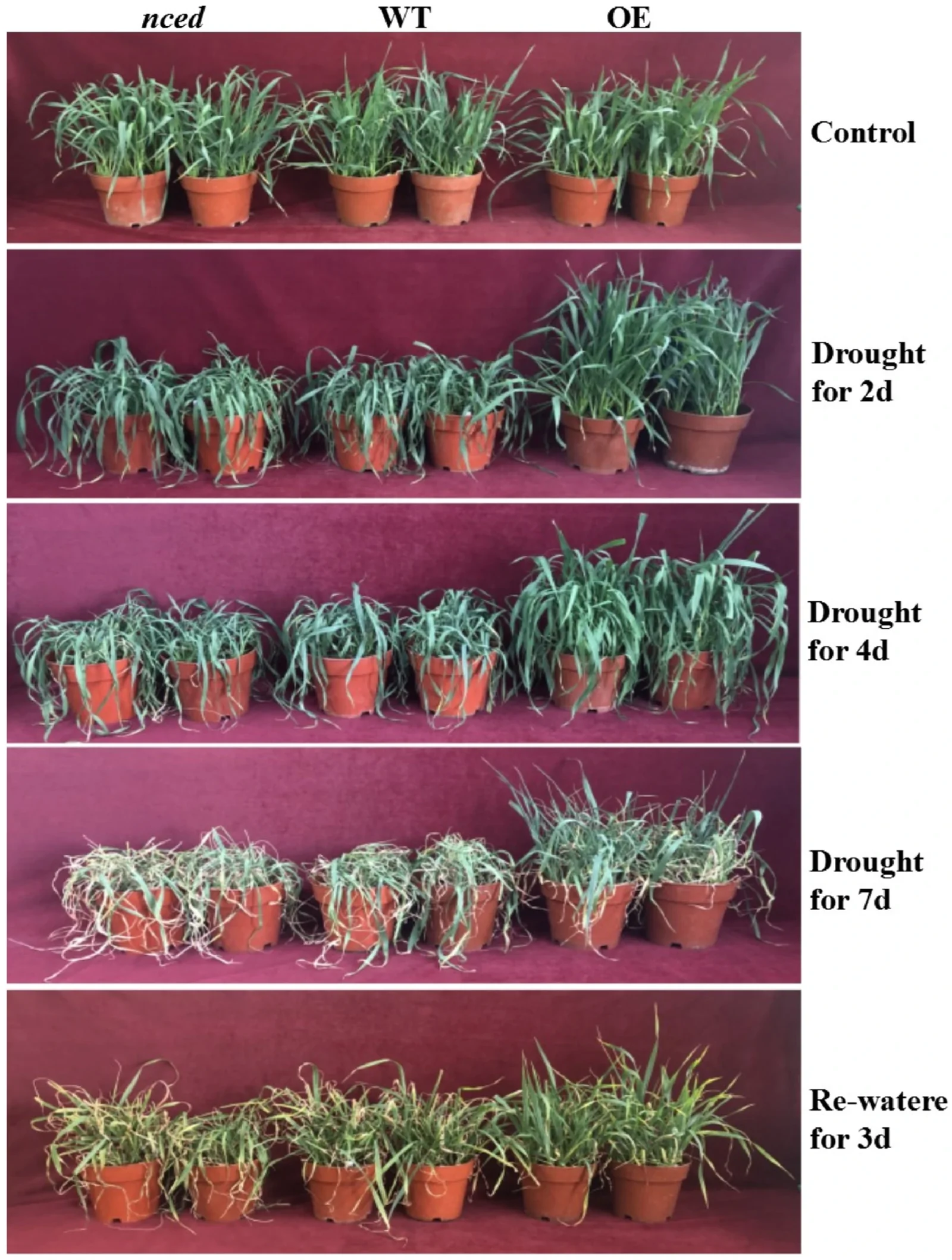
Phenotypic comparison of *TaNCED1*-overexpressing (OE), CRISPR-edited knockout (*nced*), and wild-type (WT) wheat seedlings under drought stress and re-watering treatments. Seedlings were grown under normal water supply (Control), followed by progressive drought treatment for 2, 4, and 7 days, and subsequent re-watering for 3 days to observe recovery performance.

For the drought tolerance assessment, uniformly grown three-week-old wheat seedlings were subjected to water withholding for 7 days. Clearly phenotypic differences were observed among different transgenic lines under drought conditions. As shown in Figure 3, no obvious morphological differences were observed between transgenic and WT plants under normal growth conditions. After 2 days of drought treatment, *nced* and WT plants displayed earlier water-deficit symptoms and began to wilt, while the OE lines exhibited stronger drought tolerance. By the fourth day of drought treatment, all tested materials showed more severe wilting symptoms, with the phenotypic divergence being most prominent at this stage. After 7 days without watering, the leaves of WT and mutant lines were largely yellow and withered, whereas OE seedlings retained a relatively healthy growth state. One week after re-watering, OE plants recovered much faster, suggesting they sustained less drought-induced injury. Collectively, these results demonstrate that *TaNCED1* positively enhances drought tolerance in wheat seedlings, and gene knockout exerts the opposite effect.

### 2.4. TaNCED1 Regulates Relative Water Content and CAT Contents Under Drought Treatments

**Figure 4:**
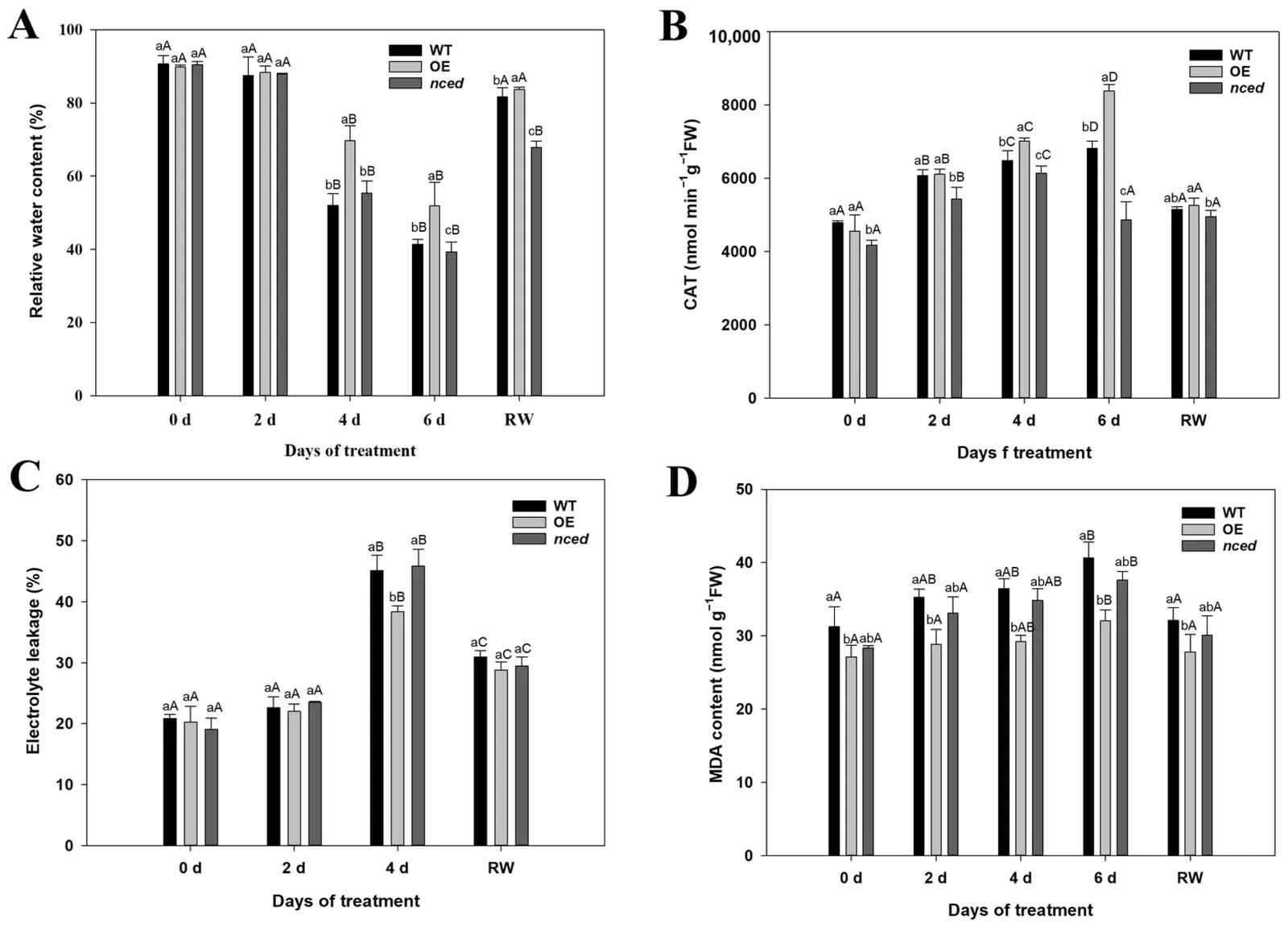
Determination of physiological indexes in OE, *nced* and WT wheat seedlings under normal and drought treatments. (**A**) Relative water content (RWC). (**B**) Catalase (CAT) activity. (**C**) Electrolyte leakage rate. (**D**) Malondialdehyde (MDA) content. Lowercase letters denote significant differences among genotypes within the same treatment time. Uppercase letters denote significant differences across sampling time points for the identical genotype. Multiple comparisons were performed by Tukey’s HSD test at *p* < 0.05. Values are mean ± SD.

Leaf relative water content (RWC) is a critical index reflecting the water retention capacity of plants. Drought stress typically triggers a reduction in leaf water content. In this assay, gradual leaf yellowing and a continuous decline in RWC were observed during drought stress treatment. As shown in Figure 4A, leaf RWC showed no significant differences among transgenic and WT plants under normal growth conditions. However, the divergence between transgenic lines and WT became evident as drought treatment progressed. After 4 days of drought exposure, all transgenic lines possessed higher RWC than WT plants. Notably, the RWC of OE lines reached 65.85%, while that of WT was only 51.99%. Following 6 days of drought stress and subsequent rehydration, OE plants still maintained higher RWC than WT. By contrast, *nced1* mutants exhibited lower RWC than the WT control. These findings indicate that the knockout mutants were more severely affected by drought stress than WT plants, while OE lines were less impacted compared with the WT control.

Catalase (CAT) acts as a core antioxidant enzyme that eliminates excess intracellular reactive oxygen species (ROS), thereby protecting cell membranes and macromolecules from oxidative damage under stress. As shown in Figure 4B, CAT activity showed no significant differences between transgenic lines and WT plants under normal conditions. In response to drought treatment, CAT activity was markedly elevated in all lines, with significant differences detected between transgenic plants and WT. On day 6 of drought stress, OE lines had the highest CAT activity, while the knockout mutants displayed lower CAT activity than WT. Taken together, CAT activity in WT and gene-edited lines were consistently lower than those in OE lines, suggesting that *TaNCED1* overexpression enhances the ROS scavenging capacity of wheat under drought stress.

### 2.5. Evaluation of the Cell Membrane Injury in Transgenic Wheat Lines

Drought stress can disrupt the integrity of plant cell walls and cellular membranes. Malondialdehyde (MDA) content and electrolyte leakage are two critical physiological indicators reflecting membrane lipid peroxidation degree and membrane permeability, which are closely correlated with plant drought tolerance. Under normal growth conditions, no significant differences in MDA and electrolyte leakage were detected among transgenic lines and WT plants. In contrast, drought stress strongly induced the upregulation of both indicators among all the plants (Figure 4C,D).

After four days of drought treatment, the electrolyte leakage of WT and the *nced* mutant plants reached 46.5% and 47.2%, respectively, whereas OE lines maintained a significantly lower level of 38.2%. After seven days of re-watering, the electrolyte leakage of OE lines decreased to 28.6%, which was still lower than that of WT plants (30.9%).

Consistent with the electrolyte leakage results, MDA content increased rapidly in response to drought stress. After 4 days of drought treatment, MDA levels in OE and gene-edited plants were 0.8-fold and 0.96-fold those of the WT control, respectively. On the sixth day of drought stress, the corresponding values were 0.78-fold and 0.93-fold relative to WT. A similar trend was maintained during the re-watering period. Overall, OE lines accumulated significantly less MDA than WT plants under drought and recovery conditions. These results suggest that *TaNCED1* effectively maintains cell membrane stability, alleviates drought-induced membrane oxidative damage, and ultimately improves wheat drought tolerance.

### 2.6. The Impact of Drought Stress on the Osmotic Regulatory Substances

**Figure 5:**
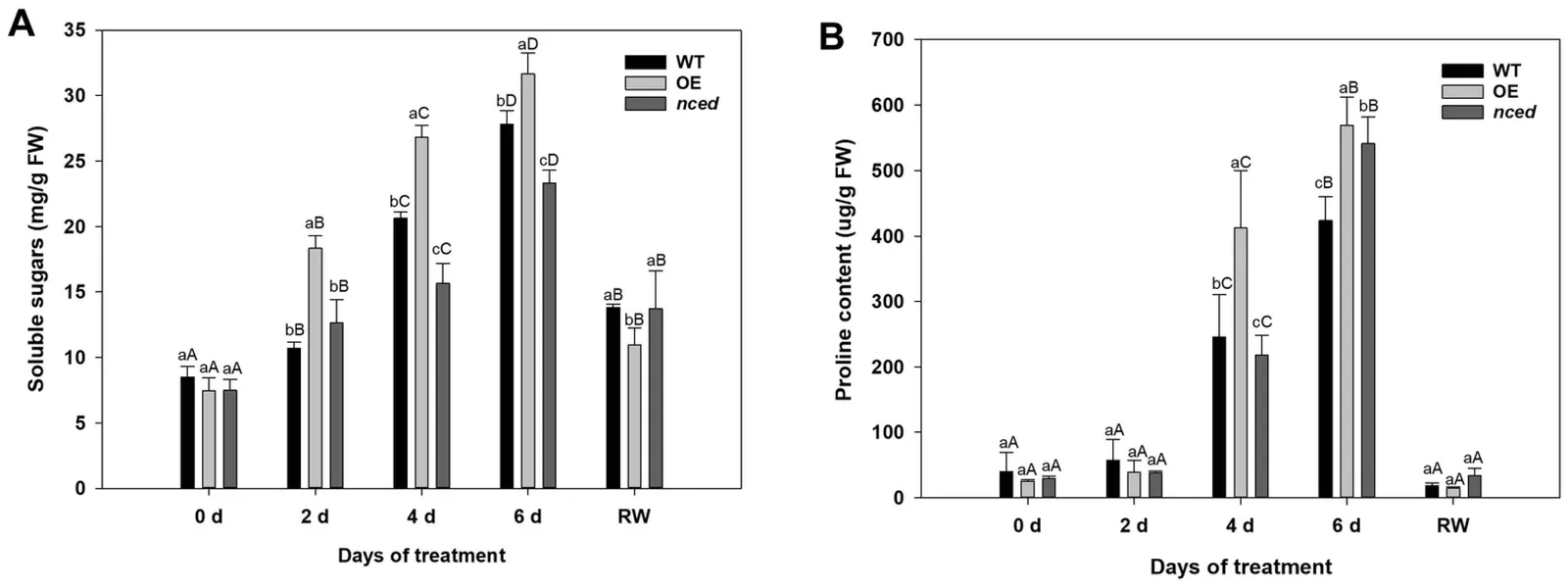
Changes in soluble sugar and proline in different wheat lines under control and drought stress. (**A**) Soluble sugar content; (**B**) proline content. Lowercase letters denote significant differences among genotypes within the same treatment time. Uppercase letters denote significant differences across sampling time points for the identical genotype. Multiple comparisons were performed by Tukey’s HSD test at *p* < 0.05. Values are mean ± SD.

Soluble sugars and proline are core osmolytes that preserve cellular turgor and osmotic homeostasis, enabling plants to adapt to drought stress. To assess the osmotic adjustment capacity of different wheat genotypes, we quantified the leaf contents of total soluble sugars and proline. Under well-watered conditions, the accumulation of soluble sugars and proline was comparable among WT and transgenic lines (Figure 5). In response to drought stress, the levels of both osmolytes continuously increased in all plants and reached their maximum values on the sixth day of drought treatment.

Throughout the entire drought stress period, the OE lines accumulated higher contents of soluble sugars and proline compared with the other wheat plants. Specifically, significantly greater soluble sugar accumulation was detected in OE seedlings on the second and fourth days during the drought stress, whereas *nced1* knockout lines exhibited lower soluble sugar accumulation than WT. After seven days of re-watering, osmotic substance contents in all plants gradually returned to normal levels, yet OE lines still retained the highest soluble sugar concentration. These results showed that *TaNCED1* overexpression promotes the accumulation of osmotic regulatory substances, enhances the osmotic adjustment ability, and thereby improves the plant tolerance to drought stress.

### 2.7. Transcriptome Analysis of TaNCED1 Target Genes in Response to Drought Stress

To investigate the possible molecular mechanisms of *TaNCED1* gene in regulating the drought stress tolerance in transgenic wheat, RNA-seq analysis was performed using the *TaNCED1* transgenic plants and WT plants. In the above experiments, the overexpression of *TaNCED1* confers drought stress-tolerance at the seedling stages, while loss-of-function *nced* mutants displayed altered stress performance under drought stress. The transcriptomic profiling of *TaNCED1* OE lines and WT plants under well-watered and water-deficit conditions were compared.

**Figure 6:**
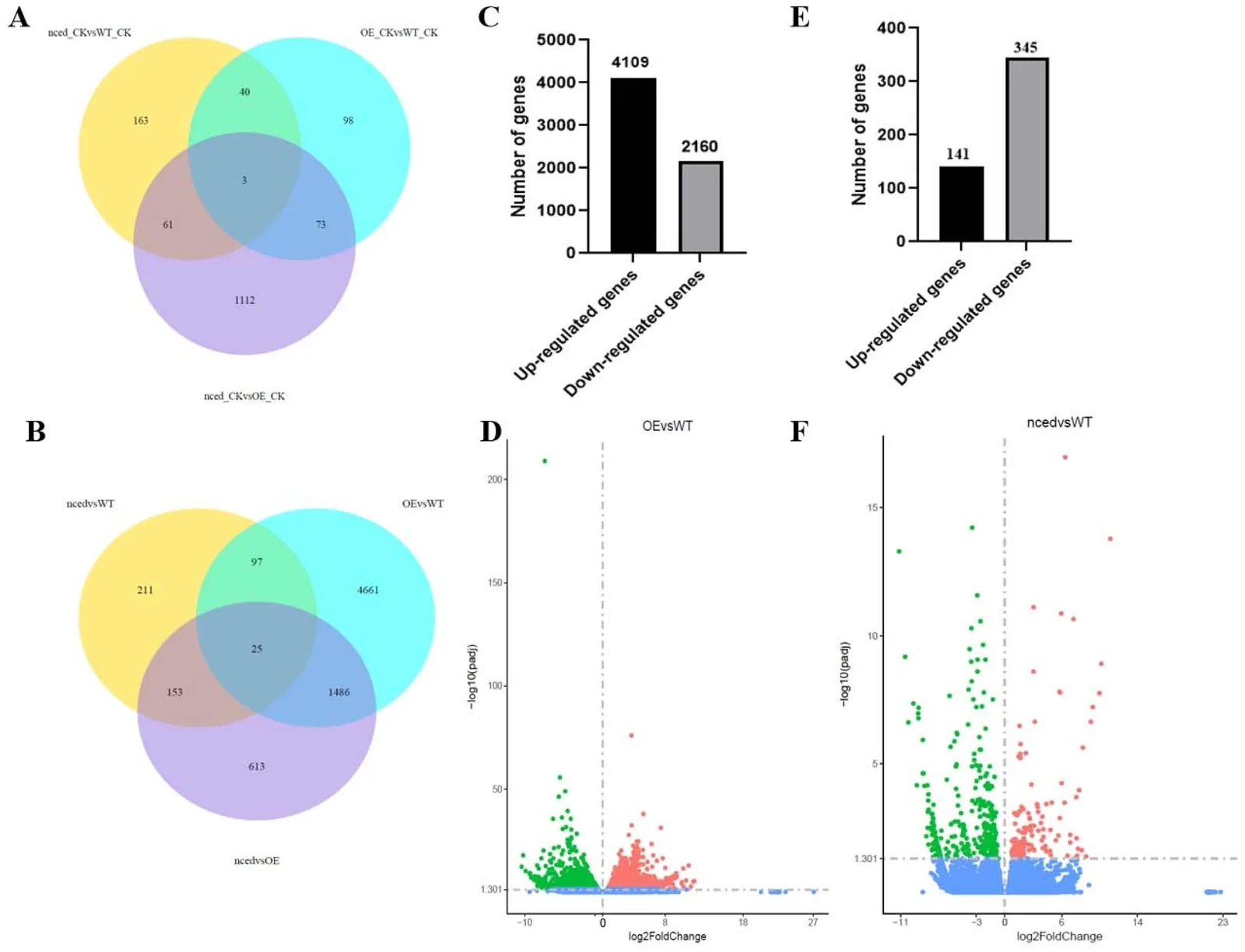
Overview of in *TaNCED1*-OE, *nced* mutant and WT wheat under control and drought conditions based on transcriptome analysis. (**A**,**B**) Venn diagrams showing the shared and unique DEGs among three comparison groups under normal control (**A**) and drought stress (**B**). (**C**,**E**) Number statistics of DEG. (**D**,**F**) Volcano plots of DEGs for OE vs. WT and *nced* vs. WT, respectively. In volcano plots, green dots represent downregulated genes, red dots represent upregulated genes, and blue dots indicate genes with no significant expression change. The light gray vertical dotted line indicates log_2_FoldChange = 0, and the light gray horizontal dotted line indicates *padj* = 0.05 (−log_10_(*padj*) = 1.301).

**Figure 7:**
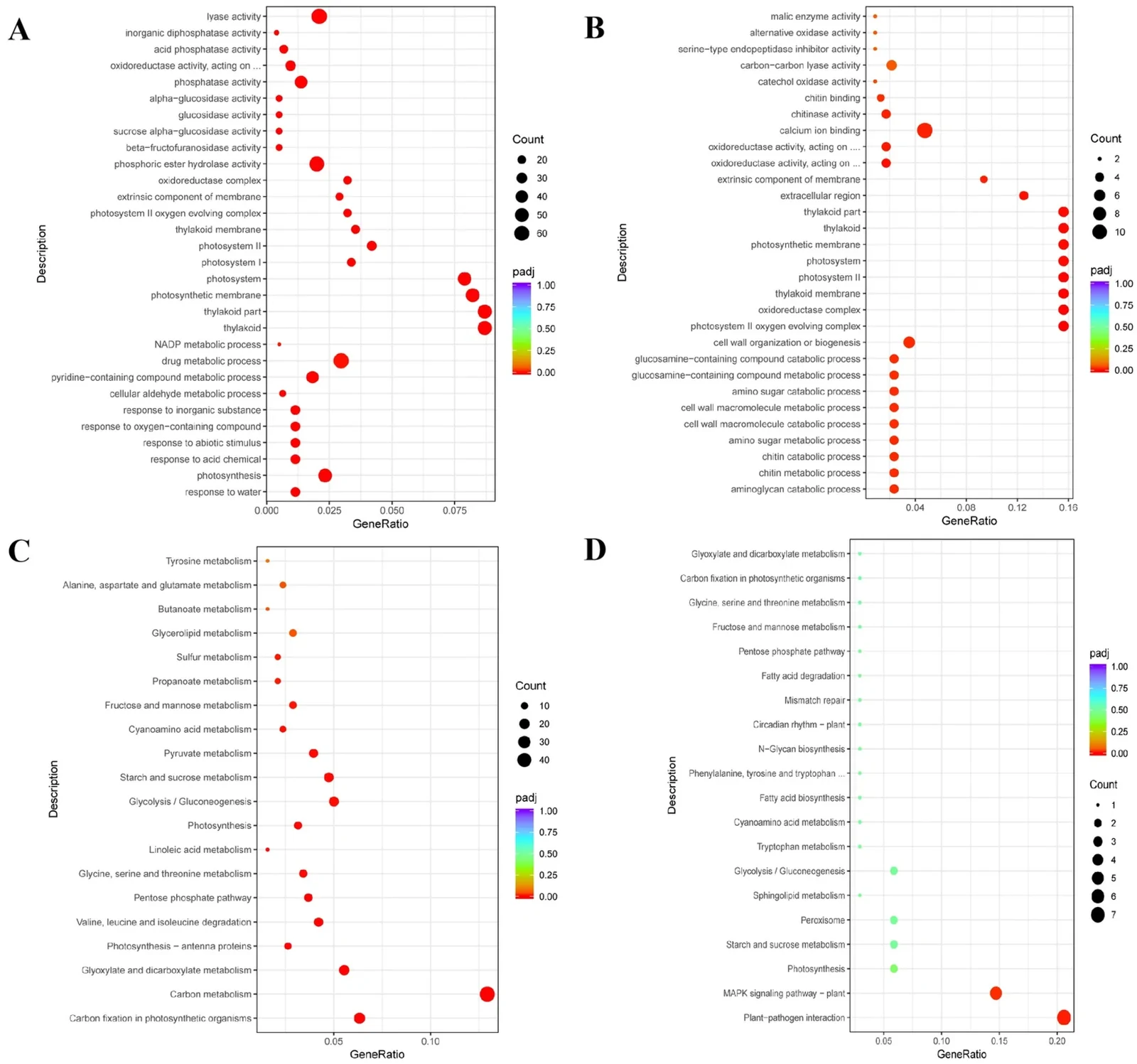
Top GO functional enrichment terms and KEGG enrichment analyses of differentially expressed genes (DEGs) from OE vs. WT and *nced* vs. WT comparisons. (**A**) GO enrichment analyses of DEGs between OE and WT. (**B**) GO enrichment analyses of DEGs between *nced* and WT. (**C**) KEGG enrichment analyses of common DEGs between OE and WT. (**D**) KEGG enrichment analyses of DEGs between *nced* and WT.

Under drought stress, a total of 6269 differentially expressed genes (DEGs) were identified between the OE plants and WT plants (Fold change >2 or FC < 0.5, *p* < 0.01). Among these genes, 4109 genes were upregulated and 2160 genes were downregulated (Figure 6). Gene ontology (GO) analysis showed that the DEGs were mainly enriched in stress-related biological processes and cellular components, such as response to water, response to abiotic stimulus, and response to oxygen-containing compounds (Figure 7A). Meanwhile, terms related to oxidoreductase complex were also significantly enriched, suggesting that *TaNCED1* overexpression extensively activates stress response and redox metabolism in wheat.

Unlike OE lines, nced mutants showed a marked reduced number of DEGs when compared with WT plants under drought conditions. In total, 141 genes were upregulated and 345 genes were downregulated in the *nced* mutants compared to WT plants. The distribution of the upregulated and downregulated genes is presented using a volcano plot (Figure 6). GO enrichment analysis revealed that the DEGs were mainly associated with calcium ion binding, oxidoreductase activity, oxidoreductase complex, cell wall organization or biogenesis and amino sugar catabolic process (Figure 7B). These results implied that the knockout of *TaNCED1* mainly disturbs cell wall structure, ion homeostasis and carbohydrate catabolism during drought stress.

KEGG pathway analysis was subsequently performed to clarify the core metabolic and signaling pathways regulated by *TaNCED1. TaNCED1*-OE plants relative to WT were enriched in carbon metabolism, starch and sucrose metabolism, and photosynthesis (Figure 7C). These pathways are essential for energy supply and carbohydrate accumulation, which help maintain normal growth and metabolism under drought. On the other hand, DEGs from *nced* mutants were predominantly enriched in the MAPK signaling pathway, starch and sucrose metabolism, peroxisome and so on (Figure 7D). The MAPK cascade is a core signal transduction module for plant abiotic stress responses, and the peroxisome pathway is critical for reactive oxygen species scavenging.

These transcriptome changes implied that *TaNCED1* could be involved in multiple regulatory networks that coordinate diverse signaling cascades, metabolic pathways and cellular processes, and integrates multiple regulatory networks to enhance drought tolerance in wheat drought resistance under stress treatment.

## 3. Discussion

Common wheat (*Triticum aestivum* L.) is one of the most widely cultivated and essential staple cereal crops worldwide. However, drought stress severely suppresses wheat growth and drastically reduces grain yield, posing a major threat to wheat production [16]. Improving drought adaptability and yield stability under water-deficit conditions has therefore become a core goal of wheat resistance breeding. Nevertheless, conventional breeding and marker-assisted selection approaches are time-consuming and labor-intensive. In contrast, genetic engineering serves as an efficient and powerful strategy for dissecting gene function and improving plant abiotic stress tolerance, providing crucial technical support for crop stress resistance improvement [17,18,19].

9-cis-epoxycarotenoid dioxygenases (NCEDs), serving as the rate-limiting enzymes in abscisic acid (ABA) biosynthesis, play a pivotal role in regulating plant growth and development, as well as responses to abiotic stresses. *NCED* genes exist as a gene family in plants. Numerous previous studies have confirmed that distinct *NCED* paralogs derived from a wide range of plant species and crop varieties exert positive regulatory effects on plant stress resistance. Different *NCED* family numbers isolated from monocots, dicots and various crop cultivars can effectively boost plant tolerance to multiple abiotic stresses such as drought, salt and heat damage [20,21,22,23,24,25,26].

The first *NCED* gene was cloned from common wheat, which resulted in the preliminary name *TaNCED1* with closest correlation with HvNCED1 [14]. Then the second *NCED* family gene designated as *TaNCED2* was characterized in 2016 [27]. Then, 13 *TaNCED* members in hexaploid wheat were identified by multi-omics characterization [28], and a comprehensive genomic atlas of the wheat *NCED* gene family was formed, detailing its evolutionary history, regulatory architecture, and functional diversification [29]. Nevertheless, comprehensive research on the biological functions and stress regulatory networks of the wheat *NCED* gene family is still lacking. Previous heterologous expression assays have revealed that wheat *TaNCED1* enhances drought tolerance in transgenic tobacco [14]. However, its endogenous physiological function and molecular regulatory mechanism in wheat remain unclear. In the present study, two types of *TaNCED1* transgenic wheat materials, including overexpression (OE) and CRISPR/Cas9-mediated knockout lines, were generated via *Agrobacterium*-mediated transformation to systematically elucidate the native function of *TaNCED1* in wheat drought response.

Drought stress triggers a series of physiological and biochemical alterations in plants, ultimately disrupting normal growth and metabolism. Plants respond and adapt to drought stress by modulating various physiological and molecular processes [30,31,32]. In this study, multiple drought-resistant physiological indices were comprehensively determined in WT and transgenic wheat lines under both normal and drought conditions. Physiological phenotypic analyses demonstrated that *TaNCED1* overexpression significantly improves wheat drought tolerance at the physiological level. Under drought stress, OE lines displayed better growth performance. Thus, *TaNCED1* function as a positive modulator of wheat drought tolerance through multi-pathways at the cellular and physiological levels. For one hand, overexpression of *TaNCED1* significantly strengthens leaf water retention capacity to slow leaf water loss under water-deficit conditions, alleviating the adverse impacts of dehydration with higher relative water content. *TaNCED1* markedly upregulates the CAT activity, which efficiently scavenges overproduced reactive oxygen species (ROS) triggered by drought [33,34]. Additionally, reducing the accumulation of MDA and the electrolyte leakage could protect lipid bilayer structures and sustain the structural integrity and functional stability of cell membranes [35]. On the other hand, *TaNCED1* facilitates accumulation of osmoprotectants, including soluble sugars and proline content, which could effectively lower cellular osmotic potential and enhance osmotic adjustment capacity [36,37]. In contrast, the knockout lines displayed aggravated drought damage, impaired ROS scavenging capability, disrupted osmotic balance compared with WT plants, and significantly reduced drought tolerance, further verifying the positive regulatory role of *TaNCED1* in wheat drought adaptation.

In the present study, the knockout plants displayed normal growth phenotypes with no obvious morphological changes under normal growth conditions. No significant differences were observed in seedling establishment and mature agronomic traits compared to wild-type controls [38]. This may indicate obvious functional redundancy among different *TaNCED* homologs in common wheat [27,28,29]. However, the precise upstream signaling pathways and downstream regulatory modules that constitute the full gene regulatory network of this target gene needs further investigation.

Transcriptomic profiling further uncovered the molecular mechanisms of *TaNCED1*-mediated drought tolerance. Functional enrichment analysis indicated that differentially expressed genes between OE and WT were predominantly enriched in photosynthesis, carbon metabolism, and starch and sucrose metabolism pathways. This suggests that *TaNCED1* positively modulates photosynthetic efficiency and carbohydrate metabolism, maintaining material and energy homeostasis to help wheat adapt to drought environments. Conversely, DEGs identified in *nced1* knockout mutants were mainly enriched in the MAPK signaling pathway and peroxisome metabolism. The loss of *TaNCED1* function impaired ABA-dependent stress signal transduction and blocked downstream drought-responsive regulatory cascades, thereby increasing wheat sensitivity to drought stress.

Collectively, this study confirms that *TaNCED1* acts as a positive regulator of wheat drought tolerance. As a key gene in ABA biosynthesis, *TaNCED1* integrates ABA-mediated stress signaling with plant primary metabolism, coordinating antioxidant defense, osmotic regulation, photosynthetic carbon metabolism, and stress signal transduction to synergistically enhance wheat drought resistance. These findings clarify the endogenous biological function of *TaNCED1* in wheat drought response at the physiological and transcriptomic levels.

This work elucidates the vital role and preliminary regulatory mechanism of *TaNCED1* in wheat drought tolerance, providing valuable genetic resources and a theoretical basis for molecular breeding of drought-resistant wheat. Future studies will focus on exploring the upstream regulatory factors and downstream target genes to further elaborate the detailed molecular regulatory network and provide reliable gene resources and theoretical reference for drought-resistant wheat molecular breeding.

## 4. Materials and Methods

### 4.1. Vector Construction and Agrobacterium-Mediated Wheat Transformation

The binary vector pLGY02 preserved in our lab was used for the construction of the overexpression vector. Its T-DNA region harbored a constitutive maize ubiquitin promoter, a nos terminator and a hygromycin resistance gene. For the overexpression vector construction, the full-length coding sequence of *TaNCED1* (JQ772528) was sub-cloned into pLGY02 and driven by the ubiquitin promoter (Figure 1A).

To obtain *nced1* mutants, a CRISPR/Cas9 editing vector was constructed. The sgRNA targeting the conserved region of the *TaNCED1* gene was designed with the help of the website tools CRISPRdirect (http://crispr.dbcls.jp/) (accessed on 12 September 2018) and CRISPOR (http://crispor.tefor.net/) (accessed on 12 September 2018). The oligonucleotide primers used are listed in Table S1. The sgRNA was driven by the TaU3 promoter and the Cas9 was driven by the maize ubiquitin promoter to create the CRISPR/Cas9 construct with the binary vector pBUE411 [38] (Figure 1B).

After the three vectors were constructed and confirmed by Sanger sequencing, they were transformed into EHA105. Transgenic wheat plants were produced by *Agrobacterium*-mediated transformation of immature embryos of the common wheat cultivar Fielder [15].

### 4.2. Molecular Characterization of the Transgenic Wheat Plants

To isolate the positive transgenic wheat plants, PCR analysis was performed by amplifying the *hyg* gene and QuickStix™ Kit (EnviroLogix, Portland, ME, USA) was used for Bar detection. Genomic DNA was extracted from the leaves of the seedlings by the DNAquick Plant System (Tiangen, Beijing, China). To isolate the positive transgenic wheat plants, PCR analysis was performed by amplifying the *hyg* gene for the *TaNCED1*-overexpressing lines. For the CRISPR/Cas9-mediated gene editing plants, transgenic plants were identified with the Enviologix QuickStix kit for Bar protein. Specific primers were designed for PCR amplification, and the Hi-TOM Gene Editing Detection Kit was used for subsequent detection of mutation [39]. Primers used in the studies are listed in the Table S1. Amplified DNA fragments of target genes were sequenced and aligned with BioEdit program which was used to analyze the nucleotide sequencing results. Indels, including insertions and deletions, occurring at the targeted sites of the target genes were considered mutations.

### 4.3. RNA Isolation and qRT-PCR

To investigate the expression levels of the *TaNCED1* gene in the transgenic plants, qRT-PCR were performed. Total RNA was isolated using the RNAprep Pure Plant kit (Tiangen, Beijing, China) from leaves of the transgenic plants at three-leaf stage. First-strand cDNA was synthesized using a PrimeScript First-strand cDNA Synthesis kit (Takara, Dalian, China). RT-qPCR was performed with the Roche 480 real-time PCR system (Roche, Mannheim, Germany) with TranStart Top Green qPCR SuperMix (Transgene, Beijing, China). The program was as follows: 95 °C for 5 min, followed by 40 cycles of 95 °C for 15 s and 56 °C for 30 s, and 72 °C for 30 s. The fold changes were calculated using the 2^−ΔΔCt^ method using *TaActin* gene as reference [40]. The primers used for RT-qPCR were listed in Table S1. Each experiment included three biological samples, each sample with three technical replicates.

### 4.4. Plant Materials and Stress Treatment

Phenotypic analyses were performed using homozygous T3 seeds of the transgenic lines. For the drought stress tolerance treatment, seeds from transgenic lines and WT plants were sown into the flowerpots with the same amount of soil. When the plants grew to the five-leaf stage, seedlings with uniform growth status were chosen and subjected to drought stress treatment. The plants were stressed for 7 d by withholding of water until significant wilting differences were observed between transgenic and WT plants. The degree of drought damage was determined by visual symptoms of foliage wilt. Photographs were taken and the plants were re-watered for 1 week for recovery. At the drought stress treatment stage, leaf samples were collected at five time points (stress for 0, 2, 4, 6 d and recovery for 7 d) and a series of relative physiological parameters were determined, such as the relative water content, electrolytic leakage, MDA, proline, soluble sugar and CAT activities. All experiments were performed in three independent biological replicates, with at least five plants per replicate.

### 4.5. Measurement of RWC, the Electrolytic Leakage, MDA, Total Soluble Sugar, Proline and Antioxidant Enzyme Activities

The leaves from the same position of seedlings were collected from wheat plants under normal and drought stress treatment conditions. RWC, MDA and the electrolytic leakage were performed as previously described [37]. For the measurement of total soluble sugar (Cat. No.: KT-2-Y), proline contents (Cat. No.: PRO-2-Y), and the enzyme activities of CAT (Cat. No.: CAT-2-Y), they were determined using commercial assay kits from Suzhou Comin Biotechnology Co., Ltd. (Suzhou, Jiangsu, China) by the spectrophotometric method.

### 4.6. RNA-Seq Assays

To better understand the regulation mechanism of *TaNCED1* in response to drought stress, transcriptome analysis of WT and transgenic plants were performed by RNA-Seq. Leaf tissues of the transgenic and WT seedlings were then collected for RNA-seq analysis with three replicates. Total RNA of each sample was isolated and sent to Novogene company. Library preparation and RNA sequencing were performed according to the manufacturer’s instructions. RNA-seq were analyzed as previously described. Genes with a corrected *p* < 0.01 and an absolute fold change >2 were regarded as significantly differentially expressed. Gene ontology (GO) and KEGG pathway enrichment analysis of significantly up- or downregulated genes were performed and used for evaluating gene function-related categories.

### 4.7. Statistical Analysis

All experiments were carried out with a minimum of three biological replicates to ensure reproducibility. Statistical analyses were performed separately for each sampling time point using one-way ANOVA, followed by Tukey’s HSD test at a significance level of *p* < 0.05. Two sets of comparisons were conducted within each time point: comparisons between the control group and drought, re-watering treatments, as well as comparisons among different genotypes (WT, OE, nced knockout lines). Data are presented as mean ± standard deviation (SD). Different double letters above bars indicate significant differences.

## 5. Conclusions

This study systematically characterized the function and regulatory mechanism of *TaNCED1* in wheat drought tolerance using overexpression and CRISPR/Cas9 knockout transgenic materials. *TaNCED1* positively regulates wheat drought resistance at the physiological and molecular levels, which improves water retention, enhances antioxidant defense, maintains cell membrane stability, and promotes osmotic adjustment. This work provides promising genetic resources for drought-resistant wheat molecular breeding.

## Data Availability

The original contributions presented in the study are fully included in the article and its Supplementary Materials. Further inquiries can be directed to the corresponding authors.

## Supplementary Materials

The following supporting information can be downloaded at: https://www.mdpi.com/article/10.3390/plants15162415/s1, Table S1: Primers for molecular characterization in transgenic wheat plants.
