# Supplementary material for: Functional Characterization of TaNCED1 in Regulating Drought Stress Tolerance in Transgenic Wheat
Source: Plants (Basel). 2026 Aug 7;15(16):2415. doi: 10.3390/plants15162415 (PMC13517013; doi:10.3390/plants15162415)
Supplement: Supplementary file 1 [file plants-15-02415-s001.zip › plants-4408916-supplementary.pdf]

Table S1. Primers for molecular characterization in transgenic wheat plants

Target gene	Oligo sequence (5'-3')	Applied to experiment
Hyg-F	CGAGTACTTCTACACAGCCATC	PCR amplification
Hyg-R	GTCTGTCGAGAAGTTTCTGATCG	PCR amplification
NCED1-qF	TCATCGCCGTCCACCAGG	qRT-PCR
NCED1-qR	GGGCCGCTCCAGGACATT	qRT-PCR
Actin-qF	GCCACACTGTTCCAATCTATGA	qRT-PCR
Actin-qR	TGATGGAATTGTATGTCGCTTC	qRT-PCR
NCED1-HF	ggagtgagtacggtgtgcCGGCCTCGTCTACTTCAAC	Hi-TOM detection
NCED1-HR	gagttggatgctggatggTACTTGAGGTACGGCTTCT	Hi-TOM detection
